# A Cross-Sectional Pilot Analysis of Downregulated Circulating MicroRNAs in Laryngeal Cancer

**DOI:** 10.3390/biomedicines13040830

**Published:** 2025-03-31

**Authors:** Crina Oana Pintea, Delia Berceanu Vaduva, Edward Seclaman, Nicolae Constantin Balica, Kristine Guran, Delia Ioana Horhat

**Affiliations:** 1 Doctoral School, “Victor Babes” University of Medicine and Pharmacy Timisoara, Eftimie Murgu Square 2, 300041 Timisoara, Romania; crina.pintea@umft.ro (C.O.P.); guran.kristine@umft.ro (K.G.); 2 Department IX, Discipline of Otolaryngology, “Victor Babes” University of Medicine and Pharmacy Timisoara, Eftimie Murgu Square 2, 300041 Timisoara, Romania; balica@umft.ro (N.C.B.); horhat.ioana@umft.ro (D.I.H.); 3 Discipline of Microbiology, Faculty of Medicine, “Victor Babes” University of Medicine and Pharmacy Timisoara, Eftimie Murgu Square 2, 300041 Timisoara, Romania; 4 Department of Biochemistry and Pharmacology, “Victor Babes” University of Medicine and Pharmacy Timisoara, Eftimie Murgu Square 2, 300041 Timisoara, Romania; eseclaman@umft.ro; 5 Center for Complex Networks Science, “Victor Babes” University of Medicine and Pharmacy Timisoara, Eftimie Murgu Square 2, 300041 Timisoara, Romania

**Keywords:** miRNA, laryngeal cancer, oncology, otolaryngology

## Abstract

**Background and Objectives:** Despite notable advances in diagnosing and managing laryngeal cancer, the disease continues to present challenges, particularly in the advanced stages. Circulating microRNAs (miRNAs) are increasingly recognized as accessible biomarkers for cancer detection and follow-up. This exploratory study centers on identifying and evaluating miRNAs that are specifically downregulated in laryngeal carcinoma patients, aiming to clarify their clinical relevance in distinguishing pre- and post-therapeutic states. **Methods:** A total of 30 patients with laryngeal cancer provided paired blood samples before and after undergoing surgical or non-surgical treatment. To reduce variability and resource demand, each set of 10 samples was pooled into three pre-treatment groups (P1, P2, and P3) and three corresponding post-treatment groups (C1, C2, and C3). Total RNA, including miRNAs, was isolated from both plasma and exosomes, followed by qPCR-based profiling (Qiagen platform). Downregulated miRNAs were singled out through statistical comparisons using Mann–Whitney U tests; receiver operating characteristic (ROC) analyses and logistic regression were further applied to assess diagnostic utility. **Results:** Seven miRNAs demonstrated significant downregulation in the pre-treatment samples (fold changes ranging from 0.20 to 0.64, *p* < 0.05). Notably, hsa-miR-107 and hsa-let-7a-5p both showed marked reductions of approximately fivefold (*p* < 0.01), suggesting a strong association with active tumor presence. In ROC analysis, hsa-miR-107 achieved an area under the curve (AUC) of 0.78 (95% CI: 0.62–0.90) with 72% sensitivity and 74% specificity in differentiating pre- from post-treatment states. A logistic regression model incorporating downregulated candidates produced odds ratios between 0.52 and 0.64 (*p* < 0.05), pointing to their potential additive value in clinical decision-making. **Conclusions:** These preliminary findings indicate that certain miRNAs, when suppressed in circulation, may be linked to the oncogenic milieu of laryngeal cancer. Confirming these observations in larger, multicenter investigations is critical, but this pilot work underscores the promise of downregulated miRNAs as biomarkers of disease activity and potential guides to therapy response.

## 1. Introduction

Laryngeal cancer represents a significant public health concern, with approximately 180,000 new cases, with an incidence of 2.0 per 100,000 individuals reported globally in 2020, and a death rate of 0.9 per 100,000 [1,2]. The incidence of laryngeal cancer varies considerably by geographical region and is predominantly higher in Southern Europe and South America, likely influenced by regional variations in risk factors such as tobacco use, alcohol consumption, and occupational exposures [3,4]. These epidemiological trends underscore the necessity for region-specific cancer control strategies and highlight the potential of molecular biomarkers in revolutionizing the early detection and management of this malignancy [5,6].

MiRNAs, small non-coding RNA molecules involved in the post-transcriptional regulation of gene expression, have been identified as pivotal players in the oncogenesis and progression of cancer [7,8,9]. Dysregulation of miRNAs contributes to various aspects of cancer biology, including cell cycle regulation, apoptosis, and metastasis [10]. Research over the past decade has particularly focused on the dual role of miRNAs as oncogenes or tumor suppressors, with recent studies revealing distinct profiles of upregulated and downregulated miRNAs in tumor tissues compared to normal mucosa [11,12,13]. For instance, miR-21, an oncogenic miRNA, is frequently upregulated in various types of cancer and associated with poor prognosis [14], while let-7g, a tumor-suppressive miRNA, is often downregulated and correlates with advanced disease stages and shorter survival [15].

The diagnostic potential of miRNAs in laryngeal cancer is promising, given their stability in body fluids and their ability to reflect underlying tumor biology [16]. Circulating miRNAs can serve as non-invasive biomarkers for early cancer detection, disease monitoring, and prognosis [17,18]. Innovative studies have demonstrated that specific miRNA signatures can differentiate between malignant and benign lesions with high sensitivity and specificity, offering a valuable tool for supplementing traditional diagnostic methods such as laryngoscopy and biopsy [19]. Targeting miRNA pathways has shown potential in preclinical models, with strategies such as miRNA mimics to restore the function of tumor-suppressive miRNAs or miRNA inhibitors to block oncogenic miRNAs [20].

Preliminary findings on the role of microRNAs (miRNAs) in oral and laryngeal cancers underscore their potential as diagnostic and prognostic biomarkers. In oral cancer, studies have highlighted the diagnostic utility of specific miRNAs such as hsa-miR-133a-3p and hsa-miR-375-3p, which were found to be significantly downregulated in patients compared to healthy controls, offering high sensitivity and specificity for early cancer detection [21]. Further analysis has identified additional miRNAs with potential diagnostic and prognostic significance in oral cancer [22]. Similarly, in laryngeal cancer, the development of a miRNA-based decision tree model has demonstrated an ability to diagnose the disease with high accuracy using blood-based miRNA signatures [23]. Comprehensive reviews and systematic analyses have further emphasized the diagnostic and prognostic importance of miRNAs in laryngeal cancer, suggesting their integration into clinical practice for improved disease management and patient outcomes [24,25].

The choice to focus on downregulated circulating miRNAs in laryngeal cancer stems from the disease’s significant public health impact, marked by varying incidence and mortality rates across regions, and its strong association with local lifestyle factors like tobacco and alcohol use. Given miRNAs’ stability in body fluids and their crucial role in cancer biology, they present a promising avenue for non-invasive diagnostics and therapeutic targeting. This study aims to explore specific downregulated miRNAs in laryngeal cancer, hoping to provide insights that could lead to improved diagnostic methods and treatment strategies, ultimately enhancing patient management and outcomes.

## 2. Materials and Methods

### 2.1. Study Participants and Design

Thirty adult patients with histologically confirmed laryngeal cancer were recruited from the ENT Clinic in Timișoara, Romania, at the Clinical Municipal Hospital affiliated with the Victor Babes University of Medicine and Pharmacy in Timisoara. Eligibility criteria included an age range of 18 to 85 years, a minimum of one year of post-therapy follow-up availability, and provision of informed consent. Patients deemed ineligible either lacked consistent follow-up or declined blood sampling for research purposes. The participants contributed paired blood samples before and after treatment, enabling a comparative exploration of miRNA changes. Each batch of 10 samples was merged into a single pooled sample—three pre-treatment pools (P1, P2, and P3) and three post-treatment pools (C1, C2, and C3).

In this study, the decision to pool ten patients into one group for each of the three pre-treatment and three post-treatment sample batches was driven by the need to mitigate variability and enhance the statistical robustness of the miRNA analysis. This pooling strategy allowed for a more manageable and economically feasible analysis given the resource constraints often present in exploratory studies. The pre-treatment groups (P1, P2, and P3) and post-treatment groups (C1, C2, and C3) were treated as separate biological replicates during statistical analysis. This approach was adopted to account for inter-patient variability and to ensure that the observed miRNA changes were representative of the broader patient population rather than individual variations. By treating these pooled groups as replicates, the study aimed to validate the consistency of miRNA expression changes across different sets of patients, thereby strengthening the reliability of the findings regarding the impact of treatment on miRNA profiles. This methodology, while resource-efficient, introduces a limitation in the ability to capture patient-specific responses, which could be addressed in future studies by analyzing individual samples.

The study was conducted according to the guidelines of the Declaration of Helsinki and approved by the Ethics Committee of the “Victor Babes” University of Medicine and Pharmacy, Timisoara, on 6 April 2023 (approval code I-9647). All participants provided informed consent, affirming their voluntary participation and understanding of the research purposes, procedures, and potential risks involved.

In this study, “pre-treatment” refers to blood samples collected from patients prior to initiating any therapeutic intervention for laryngeal cancer, ensuring baseline miRNA profiles are documented without the influence of treatment effects. “Post-treatment” samples, on the other hand, were gathered after the patients underwent a uniform treatment protocol, which could include surgical or non-surgical options such as chemotherapy or radiation, tailored to the standard care guidelines for laryngeal cancer at the recruiting institution. These post-treatment samples were collected at a specific interval, typically 2–4 weeks following the conclusion of treatment, to evaluate the immediate impact of therapy on miRNA expression. This timeframe allows for the assessment of short-term treatment effects on circulating miRNAs, potentially reflecting changes in the tumor microenvironment or tumor burden due to the therapeutic intervention.

### 2.2. Blood Sample Collection and Processing

Clinically indicated venipuncture was performed by skilled practitioners using sterile vacutainer equipment to minimize potential adverse events. From each participant, 6 mL of blood (2 × 3 mL tubes) was collected and promptly transported to the University laboratory for processing. Plasma was separated through centrifugation at 1500× *g* for 10 min to remove cells, and the resulting supernatant was stored at −80 °C for future analysis. Exosomes were then isolated from this plasma through a sequential process involving low-speed centrifugation at 300× *g* for 10 min to remove cellular debris, followed by ultracentrifugation at 100,000× *g* for 70 min and a specialized kit-based purification to ensure a clean preparation. RNA was extracted from both the free-circulating plasma fraction and the exosomal fraction using Qiagen’s miRNA extraction kits, designed to efficiently isolate small non-coding RNAs (Qiagen, Germantown, MD, USA). This method aims to capture a comprehensive profile of miRNAs circulating in the bloodstream, thus enhancing the accuracy and utility of subsequent miRNA-based assays.

### 2.3. Laboratory Profiling of Downregulated miRNAs

Total RNA was reverse-transcribed using an miRNeasy Kit (Qiagen, Germantown, MD, USA), which is specifically designed for the efficient cDNA synthesis of mature miRNAs, ensuring that all miRNAs are equally represented in the reverse transcription process. This was followed by Quantitative real-time PCR (qPCR), conducted on a miRNA PCR Array (Qiagen, Germantown, MD, USA) in a 7900HT real-time PCR instrument (Thermo Fisher Scientific, Waltham, MA, USA). The qPCR employed customizable panels capable of detecting a broad range of human miRNAs, ensuring comprehensive coverage of miRNA expression profiles. Internal controls—such as UniSP2, UniSP4, and UniSP5—and an external spike-in (cel-miR-39) were utilized to monitor qPCR efficiency and consistency across all samples. An inter-plate calibrator (UniSP3) was employed to standardize measurements across multiple runs.

### 2.4. Statistical Analysis

Data were analyzed using SPSS Statistics version 27 (IBM Corp., Armonk, NY, USA). The Mann–Whitney U test primarily served to compare pre- and post-treatment pools, given the generally non-Gaussian distribution of miRNA data. Spearman’s rank correlation was applied for pairwise comparisons among downregulated miRNAs, investigating interdependencies that might signal shared pathways. Receiver operating characteristic (ROC) curves provided insight into diagnostic performance, quantifying sensitivity, specificity, and area under the curve (AUC). Logistic regression models further evaluated the power of downregulated miRNAs to classify samples as pre- or post-treatment, adjusting for relevant covariates where feasible. Statistical significance was defined at *p* < 0.05, with emphasis on effect sizes and confidence intervals.

## 3. Results

To identify downregulated candidates, we focused on miRNAs that displayed a decrease in expression in pre-treatment pools (P1, P2, and P3) relative to post-treatment pools (C1, C2, and C3), as detailed in Table 1. Relative expression levels were calculated using the 2^(−∆Ct)^ method, where negative fold change values signified downregulation. For additional robustness in data analysis, we utilized multiple normalization strategies provided by the Qiagen data analysis suite to confirm the reliability and accuracy of the observed changes in miRNA expression.

Table 2 outlines the downregulation of specific miRNAs when comparing pre-treatment and post-treatment samples using the Mann–Whitney U test. Notably, hsa-miR-107 exhibited a decrease in mean ∆Ct from 3.48 pre-treatment to 1.18 post-treatment, resulting in a significant fold change of 0.2 (*p*-value = 0.008). Similarly, hsa-let-7a-5p showed a reduction in ∆Ct from 2.05 pre-treatment to −0.3 post-treatment, corresponding to a fold change of 0.2 (*p*-value = 0.01). Other miRNAs, including hsa-miR-146a-5p, hsa-miR-30e-5p, hsa-miR-26b-5p, hsa-let-7c-5p, and hsa-miR-23b-3p, also demonstrated significant reductions in ∆Ct values post-treatment, with fold changes ranging from 0.13 to 0.35 and *p*-values between 0.025 and 0.045.

**Figure 1:**
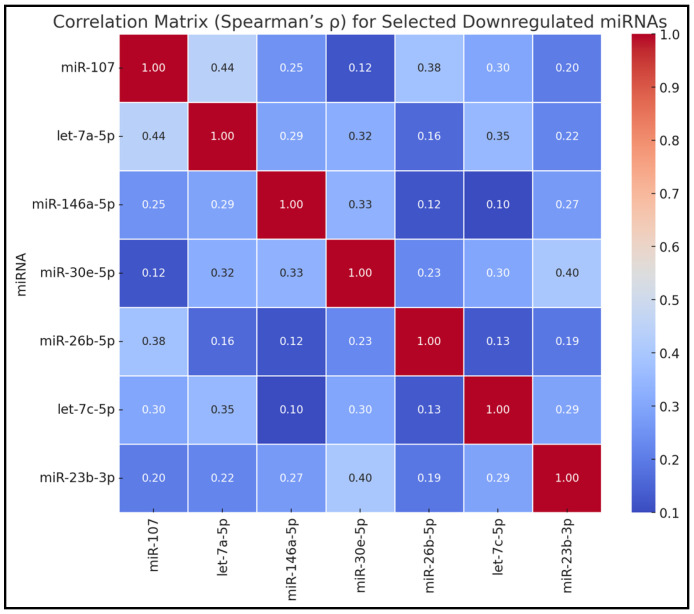
Correlation matrix for selected downregulated miRNAs.

Table 3 showcases the Spearman’s correlation coefficients among the selected downregulated miRNAs. Significant positive correlations were observed between miR-107 and let-7a-5p (ρ = 0.44, *p* < 0.05), miR-107 and miR-26b-5p (ρ = 0.38, *p* < 0.05), let-7a-5p and let-7c-5p (ρ = 0.35, *p* < 0.05), as well as miR-30e-5p and miR-23b-3p (ρ = 0.40, *p* < 0.05). Other miRNA pairs exhibited moderate correlations without reaching statistical significance, such as miR-107 with miR-146a-5p (ρ = 0.25) and let-7a-5p with miR-146a-5p (ρ = 0.29), as presented in Figure 1.

Table 4 presents the receiver operating characteristic (ROC) curve analysis for the downregulated miRNAs in distinguishing pre-treatment from post-treatment samples. miR-107 achieved the highest area under the curve (AUC) of 0.78, with a 95% confidence interval of 0.62–0.90, demonstrating 72% sensitivity and 74% specificity (*p* = 0.005). let-7a-5p followed with an AUC of 0.75, 95% CI of 0.59–0.87, 70% sensitivity, and 72% specificity (*p* = 0.01). Other miRNAs, including miR-146a-5p and miR-30e-5p, showed lower but still significant AUC values of 0.70 and 0.68, respectively. miR-146a-5p exhibited 65% sensitivity and 68% specificity (*p* = 0.03), while miR-30e-5p demonstrated 62% sensitivity and 65% specificity (*p* = 0.045).

Table 5 outlines the logistic regression model assessing the association between downregulated miRNAs and pre-treatment status. miR-107 was significantly associated with pre-treatment status, yielding an odds ratio (OR) of 0.54 (95% CI: 0.34–0.85, *p* = 0.012). Similarly, let-7a-5p demonstrated a significant association with an OR of 0.62 (95% CI: 0.40–0.94, *p* = 0.027). These ORs suggested that higher expression levels of these miRNAs were associated with lower odds of being in the pre-treatment group. The findings from the logistic regression analysis highlight the potential of miR-107 and let-7a-5p as predictive biomarkers for distinguishing pre-treatment conditions.

Table 6 summarizes the bioinformatic pathway predictions for the downregulated miRNAs, detailing their putative pathway targets, principal biological roles, and potential clinical impacts.

## 4. Discussion

### 4.1. Analysis of Findings

This study offers preliminary evidence that downregulated miRNAs can be important indicators of laryngeal cancer activity. By contrasting pooled pre-treatment and post-treatment samples, we identified several key miRNAs—most notably miR-107 and let-7a-5p—that appear markedly suppressed in active disease, then rebound once interventions are administered. The observed shifts in expression underscore the nuanced interplay between tumor cells and host regulatory mechanisms. Such miRNAs may hold value for clinicians, serving as biomarkers to detect residual disease, gauge therapeutic efficacy, or predict recurrence. In parallel, correlation insights reveal that several of these miRNAs are moderately interrelated, hinting that they might collectively modulate overlapping oncogenic pathways. Subsequent functional experimentation—ideally with in vivo models—would be beneficial in elucidating whether restoring these miRNAs can meaningfully curb malignant growth.

In prior studies, miR-107 was predicted to target CDK regulation and hypoxia response pathways, playing roles in cell cycle control and angiogenesis, and was suggested as a prognostic biomarker for disease progression [26]. let-7a-5p targeted the RAS oncogene and MAPK cascade, functioning in oncogenic checkpoints and differentiation, and was identified as a therapeutic target to restore tumor suppression [27].

Other miRNAs, such as miR-146a-5p [28], miR-30e-5p [29], miR-26b-5p [30], let-7c-5p [31], and miR-23b-3p [32], were associated with various pathways including NF-κB signaling, Notch and TGF-β pathways, Wnt signaling, and the p53 network. Their principal biological roles encompassed inflammation, apoptosis, epithelial homeostasis, and DNA damage repair. The potential clinical impacts ranged from influencing therapy-induced immunity and metastasis risk to serving as prognostic indicators for aggressive disease and targets for enhancing treatment responsiveness.

Similarly, Wang et al. [33] discovered that miRNA-26b is significantly upregulated in patients with laryngeal carcinoma compared to normal volunteers. They demonstrated that the downregulation of miRNA-26b significantly inhibits the proliferation of Hep-2 cells, an effect mediated through the targeting of ULK2 and the inactivation of the PTEN/AKT pathway. This downregulation promoted increased expression of the pro-apoptotic protein Bax and autophagy markers LC3 and p62, while reducing ULK2 and PTEN protein expressions, and increasing phosphorylated-AKT expression. In a similar manner, the study by Chen et al. [34] found that miR-150-5p is downregulated in laryngeal squamous cell carcinoma (LSCC) and that its overexpression inhibits both the proliferation and invasion of HEp-2 cells by targeting peptidyl-prolyl cis/trans isomerase NIMA-interacting 1 (PIN1). This inhibition was associated with a reduction in the expression of matrix metalloproteinases MMP-2 and MMP-9, and proteins related to epithelial–mesenchymal transition (EMT), indicating a broad impact on cancer cell aggressiveness. Both studies highlight the potential of microRNAs as therapeutic targets, but notably, they uncover contrasting patterns of miRNA expression (upregulation of miRNA-26b versus downregulation of miR-150-5p) impacting tumor biology in opposite directions through distinct molecular pathways.

The studies by Zhang et al. [35] and Song et al. [36] explore the role of microRNAs in the progression and potential treatment of laryngeal squamous cell carcinoma (LSCC), each identifying different microRNAs as key players in the disease’s pathology. Zhang et al. [35] focused on miR-206, which they found to be significantly downregulated in LSCC tissues compared to adjacent non-cancerous tissues. They demonstrated that transfection of miR-206 in LSCC cells not only reduced proliferation, migration, and invasion but also increased apoptosis, with these changes being correlated with the downregulation of vascular endothelial growth factor (VEGF). This indicates that miR-206 might act as a tumor suppressor by regulating angiogenesis and cellular invasiveness. In a similar manner, Song et al. [36] investigated miR-548ac, which they found targets the transmembrane protein 158 (TMEM158). Their results suggest that miR-548ac also acts as a tumor suppressor, primarily by inducing apoptosis in LSCC cells through the suppression of TMEM158, a protein they found to be overexpressed in laryngeal cancer tissues. Both studies underscore the therapeutic potential of targeting specific microRNAs to modulate gene expression in LSCC, suggesting these microRNAs could serve as biomarkers or therapeutic targets in the treatment of laryngeal cancer. Each study provides critical insights into how microRNAs regulate key pathways involved in LSCC progression, thus offering promising avenues for novel therapeutic strategies.

Moreover, Li et al. [37] explored the specific impact of miR-129-5p in LSCC, finding it to be upregulated in primary LSCC tumors and associated with advanced disease stages. Their experiments showed that downregulation of miR-129-5p significantly inhibited cell proliferation and migration, induced apoptosis, and caused cell cycle arrest in Hep-2 cell lines. Additionally, they demonstrated that miR-129-5p targets the adenomatous polyposis coli (APC) gene, thereby activating Wnt signaling, which promotes tumorigenesis, suggesting miR-129-5p functions as an oncogene. In a similar manner, the systematic review and meta-analysis conducted by Huang et al. [38] aggregated data from multiple studies to quantify the prognostic significance of various microRNAs in LSCC. They analyzed data involving 5307 patients and found significant associations between the levels of microRNAs such as miRNA-100, miRNA-155, miRNA-21, miRNA-34a, miRNA-195, and miR-let-7, with survival outcomes, highlighting their potential as biomarkers for disease prognosis. This systematic approach provides a broader perspective on the overall impact of microRNAs in LSCC prognosis, complementing the specific molecular insights provided by Li et al. [37]. Both studies underscore the potential of microRNAs as both therapeutic targets and prognostic biomarkers in LSCC.

The research conducted by Xu et al. [39] and Tang et al. [40] provides important insights into the molecular mechanisms influencing laryngeal squamous cell carcinoma (LSCC), specifically focusing on microRNAs (miRNAs) and their targets. Xu et al. [39] investigated the role of miR-24 in LSCC, finding that it was significantly underexpressed in LSCC cells and tissue compared to controls. They demonstrated that overexpression of miR-24 inhibited cell growth, reduced colony formation, and increased apoptosis in LSCC cells. Furthermore, miR-24 enhanced radiosensitivity in LSCC cells by promoting radiation-induced apoptosis through targeting the X-linked inhibitor of apoptosis protein (XIAP). The inverse correlation between miR-24 and XIAP expression highlights the potential of miR-24 as a therapeutic target to combat radioresistance in LSCC. In a similar manner, Tang et al. [40] utilized a comprehensive approach to identify microRNA and long non-coding RNA (lncRNA) signatures associated with the recurrence of laryngeal cancer. By analyzing data from the Cancer Genome Atlas and other databases, they constructed a competing endogenous RNA (ceRNA) network, revealing key interactions such as HCG4-miR-33b and HOTAIR-miR-1-MAGEA2, which could be linked to recurrence mechanisms. Their use of a support vector machine classifier to predict cancer recurrence with high accuracy further underscores the potential of RNA molecules as biomarkers for LSCC prognosis and recurrence. Both studies underscore the critical role of miRNAs in the regulation of gene expression related to LSCC progression and recurrence, offering new avenues for targeted therapies and diagnostic strategies.

Circulating microRNAs (miRNAs) have garnered substantial interest as non-invasive biomarkers that hold promise for revolutionizing the diagnosis and management of oral and laryngeal cancers. Given the direct accessibility of tumors in these regions to the oropharynx, the potential for liquid biopsy techniques extends beyond blood samples to include saliva, providing a highly convenient and patient-friendly diagnostic medium. Studies have demonstrated that miRNAs in saliva, serum, and plasma reflect the underlying tumor biology, offering insights into the initiation, progression, and potential metastasis of oral malignancies [41,42]. Furthermore, liquid biopsy, including circulating miRNAs, enables early tumor detection, monitoring of disease heterogeneity, and evaluation of treatment efficacy, which are crucial for improving patient prognosis and personalizing therapy strategies [43]. The integration of liquid biopsy into clinical practice could substantially enhance screening programs, especially for tumors that are often diagnosed at advanced stages due to current limitations in screening and physical examination strategies. However, the application of these advanced diagnostic tools in routine clinical settings still faces significant challenges, including the need for standardized methodologies and validation of specific miRNA biomarkers in larger, more diverse patient cohorts [41,42,43].

In assessing the biological significance of the observed miRNA fold changes ranging from 0.20 to 0.64, despite their statistical significance, it is crucial to consider their potential impact in the context of cellular and molecular biology. MiRNAs are potent regulators of gene expression, capable of influencing multiple pathways by targeting various mRNAs. Therefore, even modest alterations in their expression could exert substantial effects on critical cellular functions such as differentiation, proliferation, and apoptosis. Particularly in diseases like cancer, where miRNAs may act as oncogenes or tumor suppressors, these changes, although seemingly slight, could meaningfully affect disease progression and response to therapy. Thus, while these changes are statistically significant, their true biological impact should be evaluated through further functional assays and by integrating these findings with other omic data to elucidate the consequential effects on the cellular phenotype and clinical outcomes.

From a methodological perspective, this pilot investigation highlights both the strengths and caveats of pooling samples, as well as the value of analyzing free-circulating versus exosome-encapsulated miRNA compartments. While our results are promising, replicating them in broader, prospective trials is crucial to bolster clinical applicability. Future work might integrate data from upregulated miRNAs, protein biomarkers, and genetic mutations to build a multifaceted biomarker panel that offers a robust portrait of tumor biology. Ultimately, the downregulated miRNAs characterized here mark a substantive step toward personalized laryngeal cancer diagnosis and therapy monitoring.

Evaluating miRNA downregulation in pre-treatment and post-treatment samples provided critical insights into the dynamic changes induced by therapeutic interventions in laryngeal cancer. This approach allowed us to identify potential miRNA biomarkers that could serve as indicators of treatment efficacy, contributing to personalized medicine strategies by potentially predicting response rates and adjusting treatments accordingly.

### 4.2. Study Limitations

Several limitations frame these findings and should guide their interpretation. Firstly, the pilot nature of our study and reliance on pooled samples impose constraints on granularity, potentially obfuscating important inter-individual variation. Although pooling can efficiently highlight overarching trends, it diminishes resolution at the single-patient level. Secondly, the post-treatment sampling window (2–4 weeks) may not capture longer-term miRNA dynamics, particularly in scenarios of delayed recurrence or prolonged healing. Thirdly, we did not assess other epigenetic or molecular markers that could corroborate and expand on the observed miRNA changes—DNA methylation, for instance, might yield complementary insights. Fourthly, predicted signaling pathways remain speculative until validated through targeted molecular assays, leaving some uncertainty regarding the mechanistic underpinnings.

However, this study did not differentiate between responder and non-responder groups or incorporate other clinical outcomes such as survival rates or recurrence, which could have provided a deeper understanding of the relationship between miRNA profiles and patient-specific therapeutic outcomes. This limitation stems primarily from the exploratory nature of the study and the initial focus on establishing a foundational understanding of miRNA behavior in response to treatment, rather than on comprehensive outcome-based stratification. Future studies should aim to incorporate these elements to enhance the clinical applicability of the findings.

## 5. Conclusions

In conclusion, this pilot evaluation demonstrates that specific miRNAs exhibit notable downregulation in patients with active laryngeal cancer, returning toward baseline levels post-treatment. The suppressed expression of miRNAs such as miR-107 and let-7a-5p strongly correlates with tumor presence and likely reflects an erosion of critical tumor-suppressive pathways. Though these findings require further exploration in expanded cohorts, they underscore the promise of downregulated miRNAs as complementary biomarkers for diagnosis, prognosis, and monitoring response to therapy.

In practical terms, quantifying key downregulated miRNAs in peripheral blood may offer clinicians a minimally invasive means of assessing disease burden and therapeutic efficacy. When integrated with established clinical workflows—including imaging, endoscopy, and histopathological assessment—these miRNA signatures could fine-tune patient management by identifying high-risk individuals or those at greatest danger of relapse. In the longer view, strategies aimed at reintroducing or stabilizing suppressed miRNAs may also emerge as novel therapeutic avenues, supplementing existing surgical, chemotherapeutic, and radiotherapeutic regimens. By delineating preliminary links between miRNA loss and laryngeal cancer pathology, this investigation lays the groundwork for future research that can refine these insights into viable clinical tools.

## Figures and Tables

**Table 1:** Overview of pooled pre-treatment and post-treatment samples.

Group	Sample Origin	Number of Samples Pooled	Treatment Phase
P1	Pre-Treatment (Plasma)	10	Before Intervention
P2	Pre-Treatment (Plasma)	10	Before Intervention
P3	Pre-Treatment (Plasma)	10	Before Intervention
C1	Post-Treatment (Plasma)	10	2–4 Weeks After
C2	Post-Treatment (Plasma)	10	2–4 Weeks After
C3	Post-Treatment (Plasma)	10	2–4 Weeks After

**Table 2:** Key miRNAs downregulated in pre-treatment vs. post-treatment samples (Mann–Whitney U).

miRNA	Mean ∆Ct Pre (P)	Mean ∆Ct Post (C)	Fold Change (Pre vs. Post)	*p*-Value
hsa-miR-107	3.48	1.18	0.2	0.008
hsa-let-7a-5p	2.05	−0.3	0.2	0.01
hsa-miR-146a-5p	2.32	−0.16	0.18	0.03
hsa-miR-30e-5p	1.05	−0.48	0.35	0.045
hsa-miR-26b-5p	3.57	1.6	0.26	0.04
hsa-let-7c-5p	6.13	3.19	0.13	0.035
hsa-miR-23b-3p	3.6	0.78	0.14	0.025

**Table 3:** Correlation matrix (Spearman’s ρ) for selected downregulated miRNAs.

miRNA	miR-107	let-7a-5p	miR-146a-5p	miR-30e-5p	miR-26b-5p	let-7c-5p	miR-23b-3p
miR-107	1	0.44 *	0.25	0.12	0.38 *	0.3	0.2
let-7a-5p	0.44 *	1	0.29	0.32	0.16	0.35	0.22
miR-146a-5p	0.25	0.29	1	0.33	0.12	0.1	0.27
miR-30e-5p	0.12	0.32	0.33	1	0.23	0.3	0.40 *
miR-26b-5p	0.38 *	0.16	0.12	0.23	1	0.13	0.19
let-7c-5p	0.3	0.35	0.1	0.3	0.13	1	0.29
miR-23b-3p	0.2	0.22	0.27	0.40 *	0.19	0.29	1

* Statistically significant (*p*-value < 0.05).

**Table 4:** ROC curve analysis for downregulated miRNAs distinguishing pre- vs. post-treatment.

miRNA	AUC	95% CI	Sensitivity (%)	Specificity (%)	*p*-Value
miR-107	0.78	0.62–0.90	72	74	0.005
let-7a-5p	0.75	0.59–0.87	70	72	0.01
miR-146a-5p	0.7	0.54–0.82	65	68	0.03
miR-30e-5p	0.68	0.51–0.81	62	65	0.045

**Table 5:** Logistic regression model for pre-treatment status using downregulated miRNAs.

Variable	Odds Ratio (95% CI)	*p*-Value
miR-107	0.54 (0.34–0.85)	0.012
let-7a-5p	0.62 (0.40–0.94)	0.027
Constant	–	0.001

**Table 6:** Bioinformatic pathway predictions for downregulated miRNAs.

miRNA	Putative Pathway Targets	Principal Biological Roles	Potential Clinical Impact
miR-107 [26]	CDK regulation, Hypoxia response	Cell cycle control, angiogenesis	Prognostic biomarker for progression
let-7a-5p [27]	RAS oncogene, MAPK cascade	Oncogenic checkpoint, differentiation	Therapeutic target to restore tumor suppression
miR-146a-5p [28]	NF-κB, immune modulation	Inflammation, apoptosis	Might influence therapy-induced immunity
miR-30e-5p [29]	Notch, TGF-β	Epithelial homeostasis	Alteration linked to metastasis risk
miR-26b-5p [30]	Cyclin E, Wnt signaling	Cell cycle arrest, growth inhibition	Potential synergy with chemotherapeutics
let-7c-5p [31]	HMGA2, KRAS	Cell proliferation, metabolism	Could impact treatment responsiveness
miR-23b-3p [32]	p53 network, invasion genes	DNA damage repair, EMT	Prognostic for aggressive disease

## Data Availability

The data presented in this study are available on request from the corresponding author.
